# Case report: A case of classic hairy cell leukemia with CNS involvement treated with vemurafenib

**DOI:** 10.3389/fonc.2022.1100577

**Published:** 2023-01-12

**Authors:** Anna E. Johnson, Athul Raj Raju, Aasems Jacob, Gerhard C. Hildebrandt

**Affiliations:** ^1^ Department of Internal Medicine, University of Kentucky, Lexington, KY, United States; ^2^ Department of Hematology and Oncology, Pikeville Medical Center, Pikeville, KY, United States; ^3^ Division of Hematology and Medical Oncology, University of Missouri, Columbia, MO, United States

**Keywords:** hairy cell leukemia, vemurafenib, hairy cell, leukemia, hematologic malignancies, brain metastasis

## Abstract

Hairy cell leukemia (HCL) is a rare mature B-cell lymphoproliferative disorder and most often presents as classic hairy cell leukemia. This entity is characterized by an indolent course and the presence of the *BRAF* V600E mutation. We report the case of an 80-year-old man with a history of classical hairy cell leukemia who presented with fatigue, dizziness, shortness of breath, blurring of vision, and headache. His initial diagnosis was 9 years prior, and he received treatments with cladribine, pentostatin, and rituximab. The workup showed an elevated white blood cell count with atypical lymphocytes, anemia, and thrombocytopenia. A peripheral blood smear confirmed HCL relapse, and a magnetic resonance imaging (MRI) of the brain showed diffuse, nonenhancing masses in the supratentorial and infratentorial regions of the brain. He was initiated on treatment with vemurafenib, with improvements in his white blood cell count and a recovery of his platelet count and hemoglobin. A repeat MRI of the brain after 3 months showed complete resolution of the lesions. Vemurafenib was discontinued after 6 months, with bone marrow biopsy showing no evidence of residual hairy cell leukemia. There have only been limited reports of HCL involvement in the central nervous system in the literature. Due to the rarity of the condition, it is not clear which treatments can be effective for intracranial disease control. Our report shows the successful use of vemurafenib, resulting in complete remission of relapsed HCL with CNS involvement.

## Introduction

Hairy cell leukemia (HCL) is an indolent, mature B-cell lymphoproliferative disorder characterized by distinct clinical and pathological features. With only 600 to 800 new cases in the USA each year, this remains a rare hematologic malignancy. Most reported cases occur in middle-aged men with an average age at diagnosis of approximately 55 and male to female ratio of 4:1. Neoplastic B cells are described morphologically by their “hairy” cytoplasmic projections and accumulate in the bone marrow, spleen, and peripheral blood, with affected individuals presenting most often with symptomatic cytopenias and/or splenomegaly. Although it was first described by Bouroncle et al. in 1958, hairy cell leukemia remained a poorly understood entity, with management primarily consisting of splenectomy, which results in short-term survival (1). Outcomes were significantly improved with the use of purine analogs and anti-CD20 monoclonal antibody therapy, resulting in durable responses and remissions (2). Relapsed or refractory disease remained a treatment challenge until the identification of *BRAF* V600E mutations as the causal genetic abnormality, offering a targeted therapy for this pretreated population (3). Classic HCL (HCLc) and HCL variant (HCLv) have unique immunophenotypic and molecular differences with an overwhelming majority of HCLc harboring a *BRAF* V600E mutation and following an indolent course. *BRAF* V600E mutation is absent in HCLv, has an aggressive clinical course, and is less responsive to standard therapies. CNS involvement with either subtype of HCL has rarely been described in the literature. Treatment remains unclear in this situation and is often adopted from case reports. We describe a case of relapsed *BRAF*-mutated HCLc presenting with hyperlymphocytosis and intracranial involvement that was successfully treated with the oral BRAF inhibitor, vemurafenib.

## Case description

An 80-year-old white man with a history of HCL presented with complaints of fatigue, dizziness, shortness of breath, blurring of vision, and headache. He also had a history of diabetes, hypertension, and sleep apnea. HCL was initially diagnosed 9 years prior and treated with cladribine with partial response. He relapsed 3 years later after first-line therapy and was treated with pentostatin and rituximab. Follow-up evaluation with PET/CT and bone marrow biopsy showed no signs of residual disease. Following disease relapse 5 years later when he presented with lymphocytosis and splenomegaly, molecular testing was positive for *BRAF* V600E mutation. He was treated with cladribine and rituximab but was incompletely treated due to complications of neutropenic fever and an admission for intracranial hemorrhage.

**Figure 1:**
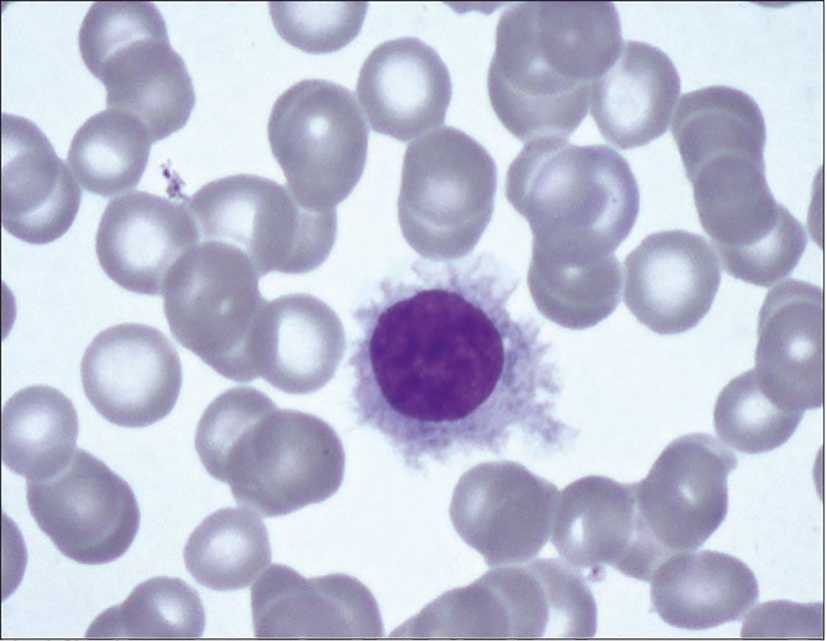
Peripheral blood smear with Wright-Giemsa staining showing hairy cell with abundant pale blue cytoplasm, slightly irregular nucleus with ground glass chromatin and hairy cell projections.

**Figure 2:**
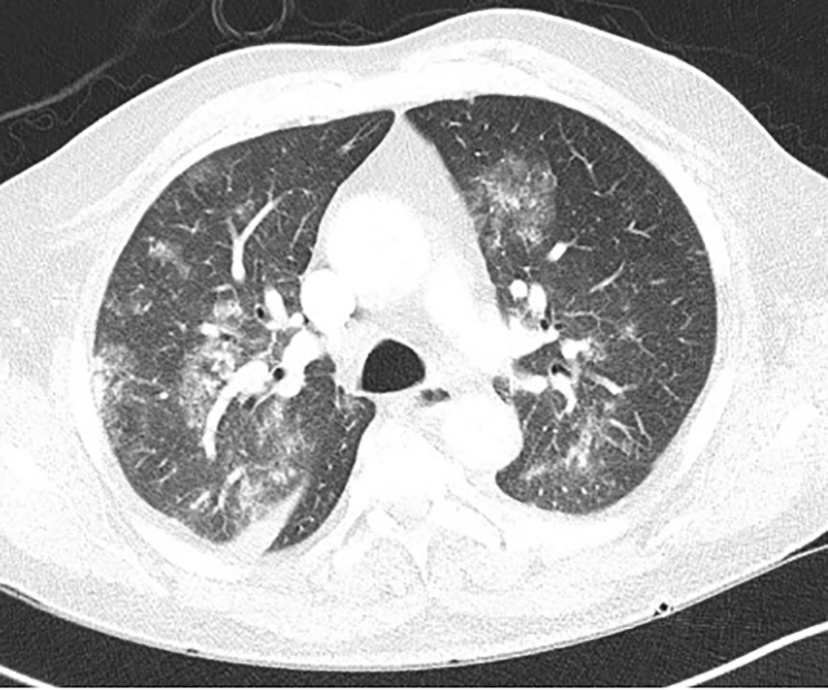
CT chest (axial) showing diffuse ground glass opacities in bilateral lung parenchyma and no radiologic evidence of pulmonary hemorrhage.

Physical examination revealed multiple petechiae and bruising in the upper and lower extremities. He had marked abdominal distension with a spleen tip palpable beyond the umbilicus. Neurologic examination showed no focal deficits. Axillary and inguinal lymph nodes were palpable. Labs showed a marked leukocytosis of 371,700 × 10^9^/L (normal: 3,500–13,000 × 10^9^/L) with 82% atypical lymphocytes, 4% segmented neutrophils, 3% lymphocytes, and 3% monocytes; hemoglobin of 6.4 g/dl (normal: 11–17 g/dl); and platelet count of 99,000 × 10^9^/L (normal: 135,000–450,000 × 10^9^/L). The patient was hospitalized with these acute findings. A peripheral blood smear showed significant lymphocytosis with oval to slightly irregular nuclei, homogenous ground-glass chromatin, and abundant pale blue cytoplasmic hairy projections, normocytic anemia with anisopoikilocytosis, and marked thrombocytopenia (**Figure 1**). A computerized tomography (CT) scan of the chest, abdomen, and pelvis showed splenomegaly of 22 cm, ground-glass opacities in bilateral upper lung fields, and small pleural effusions bilaterally. With worsening shortness of breath, increasing oxygen requirement, and the CT findings concerning leukostasis (**Figure 2**), he was started on hydroxyurea 2 g daily and underwent three sessions of leukapheresis over a period of a week with significant cytoreduction. He reported improvement in respiratory symptoms with an objective reduction in white blood count (WBC) to 89,000 × 10^9^/L. Flow cytometry on peripheral blood returned a kappa light chain monoclonal B-cell population expressing CD11c, partially dim CD10, CD103, and aberrant CD5, but without CD25 expression, consistent with relapsed HCL.

**Figure 3:**
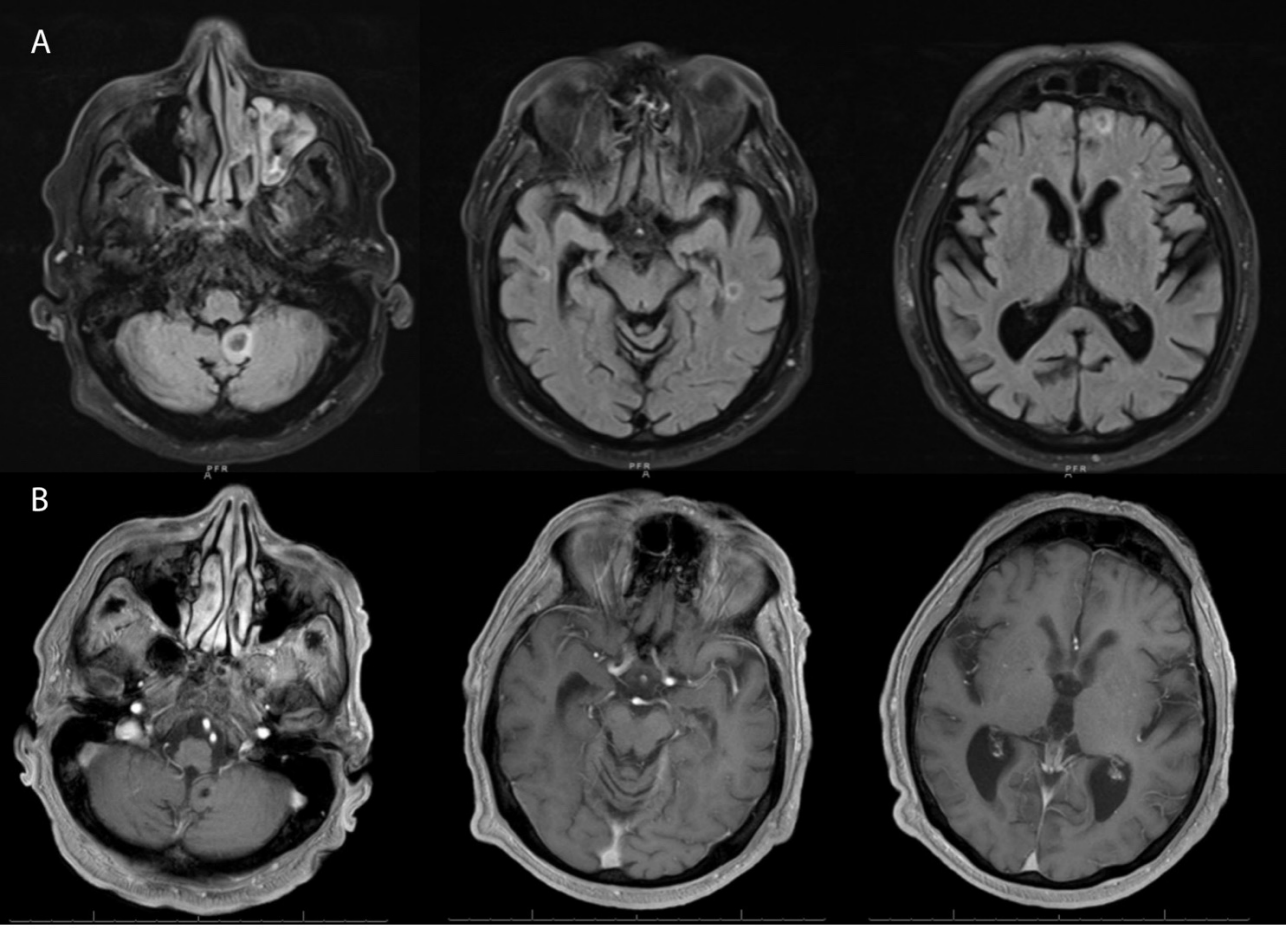
**(A)** Magnetic resonance imaging (axial section) of brain at recurrence showing numerous non-enhancing lesions (only showing index lesions) in the supratentorial and infratentorial brain with susceptibility artifact and subtle restricted diffusion of the margins and T2 hyperintense signals surrounding them, with the largest lesion in the left cerebellar vermis (14x12mm). **(B)** Follow up MRI after 4 months showing resolution of the non-enhancing lesions and only a small foci of low signal intensity noted within the posterior fossa representing chronic hemorrhage.

To identify the etiology of headache and dizziness, a CT scan of the head was done, which revealed foci of minimal hypoattenuation in both cerebellar hemispheres and periventricular white matter. This was followed by a magnetic resonance imaging (MRI) of the head with and without contrast, which showed multiple soft tissue masses in the supratentorial and infratentorial regions involving the cerebellar lobes, the left frontal and bilateral temporal lobes, with the largest lesion seen in the left cerebellum (14 × 12mm) (**Figure 3A**). A biopsy was not pursued due to the risk of the procedure and medical frailty. Extensive infectious disease evaluation was pursued with negative serology for HIV, hepatitis B and C, syphilis, toxoplasma, and fungal organisms including *Aspergillus*, *Blastomyces*, coccidiosis, histoplasma, and quantiferon TB. We initiated therapy with ibrutinib at 420 mg daily, but this was discontinued due to thrombocytopenia, epistaxis, and retinal hemorrhage after 7 days of treatment. Therapy was changed to BRAF inhibitor vemurafenib at 240 mg twice daily. Because he tolerated the treatment well and his symptoms improved, the dose was increased to 480 mg twice daily and he was discharged home.

He reported that his neurologic symptoms had resolved completely 1 month later. Labs showed a WBC of 28,400 × 10^9^/L, hemoglobin of 13.7 g/dl, and platelet count of 44,000 × 10^9^/L. An MRI of the brain, 3 months after initiation of vemurafenib, showed complete resolution of the intracranial lesions (**Figure 3B**), and abdominal imaging revealed a marked decrease in spleen size. Follow-up ophthalmologic evaluation showed resolution of leukemic retinopathy. He is planning to continue vemurafenib, with a goal to discontinue treatment once a bone marrow biopsy confirms morphologic remission

## Discussion

Historically speaking, HCLc follows a more indolent course, often not requiring treatment for extended periods of time. Once there is a treatment indication based on constitutional symptoms of fatigue and weight loss, symptomatic cytopenias, and or discomfort associated with organomegaly, first-line therapy with purine analogs results in durable remissions. With subsequent courses, however, there is the risk of toxicity and decreased efficacy. Although the central nervous involvement of HCL has been cited in the literature, it is an extremely rare entity and is often thought to be associated with a more atypical or aggressive course.

Our patient had an atypical presentation with extreme leukocytosis (371,700 × 10^9^/L) resulting in leukostasis, massive splenomegaly, and CNS involvement concerning for HCLv. Results of his flow cytometry and *BRAF* V600E mutation were most consistent with HCLc, although there was a lack of CD25 expression, which has often been associated with a more aggressive course. Given his symptoms associated with leukostasis, he was treated with cytoreductive therapy and underwent multiple sessions of leukapheresis with a brief improvement in his shortness of breath. He was started on ibrutinib based on data by Rogers et al. (4), yet this was held by our team after 1 week due to rapidly worsening thrombocytopenia and bleeding complications. Moxetumomab, an anti-CD22 immunotoxin, although a therapeutic option for relapsed and refractory HCLc, was not chosen in this case due to the availability of the drug and a lack of data in CNS disease. Based on evidence supporting oral BRAF inhibitors in relapsed or refractory BRAF-mutated HCL, he was started on vemurafenib at 240 mg twice a day, the lowest dose due to age and expected toxicity (5. He had a significant response with improvement in WBC (39,000 × 10^9^/L) and platelet count (89,500 × 10^9^/L) within 1 week of treatment. Although anti-CD20 monoclonal antibody therapy is suggested with BRAF inhibitor in the relapsed setting, we did not include this due to the high disease burden and risk of tumor lysis syndrome. Our case demonstrates the importance of evaluating any neurological symptoms in patients with HCL with imaging. A limitation in our case is that the intracranial lesions were not biopsy-proven to be involved in HCL. Due to the patient’s medical frailty and tenuous respiratory status, a brain biopsy and lumbar puncture were felt to represent significant potential harm and were therefore not pursued. An extensive infectious workup was negative, and the treatment response confirms the diagnosis.

There have been a few other case reports of CNS involvement with HCL. A reported patient from 1985 presented with CNS involvement of hairy cells in the cerebrospinal fluid (CSF) and was treated with intrathecal methotrexate with neurological improvement but had complications of *Cryptococcus neoformans* meningitis. Further treatment with alpha interferon helped in disease control (6). Another case from 1984 involved a patient who presented with leukemic meningitis, which was confirmed by morphologic exam of leukemia cells in his CSF and bone marrow. He was treated with whole brain radiation and intrathecal chemotherapy, with a resolution of his symptoms (7). More recently, a patient presented with CNS involvement and was treated with high-dose methotrexate, cladribine for 7 days, and high-dose steroids. Unfortunately, he died from gastrointestinal bleeding and sepsis (8). Another case of newly diagnosed BRAF-mutated HCLc presented with biopsy-proven multifocal CNS involvement and lymphocytosis (35.4 × 10^9^/L). He was treated successfully with cladribine and rituximab, resulting in the resolution of intracranial lesions. This patient eventually relapsed and was treated with vemurafenib, achieving a complete response (9). There is only one other case of relapsed *BRAF* V600E-mutated HCL presenting with pancytopenia and CNS involvement, initially thought to be mantle cells. This patient was treated with high-dose cytarabine, intrathecal methotrexate, and rituximab, achieving a partial response. He had immediate progression of his disease and was again treated with rituximab and intrathecal methotrexate, which stabilized the disease. At subsequent progression, he was successfully treated with vemurafenib at 960 mg twice daily, which resulted in the resolution of intracranial lesions (10).

This case demonstrates that vemurafenib can be effective at lower doses than originally suggested and without the addition of anti-CD20 monoclonal antibody therapy. Our patient was treated with vemurafenib at 480 mg twice a day; however, based on the trajectory of his response, we suspect the dose likely would have been effective at 240 mg twice a day. Additionally, there has been previous concern and speculation that vemurafenib may not be effective in treating CNS disease due to its molecular weight and inability to effectively pass the blood–brain barrier although recent literature has supported its effectiveness (11–13). This case clearly demonstrates the efficacy of vemurafenib in the setting of central nervous system disease.

Patients receiving therapy should be counseled on potential side effects such as fatigue, alopecia, rash and photosensitivity, nausea, and arthralgias. The optimal duration of oral vemurafenib is thought to be approximately 16 to 18 weeks based on prior research, with a median time to response of 8 to 12 weeks. A longer course of therapy has not been shown to improve the duration of response, with highly effective response rates and some complete remissions observed within the recommended course of treatment (14).

## Data availability statement

The original contributions presented in the study are included in the article/supplementary material. Further inquiries can be directed to the corresponding authors.

## Ethics statement

Written informed consent was obtained from the individual for the publication of any potentially identifiable images or data included in this article.

## Author contributions

AJo, AR, AJa and GH were involved in conceptualization, manuscript preparation, editing and review of the manuscript. All authors agree to be accountable for the content of the work. All authors contributed to the article and approved the submitted version.
